# Hypothalamic Astrocytes Respond to Gastric Mucosal Damage Induced by Restraint Water-Immersion Stress in Rat

**DOI:** 10.3389/fnbeh.2016.00210

**Published:** 2016-11-01

**Authors:** Haiji Sun, Ruisheng Li, Shiguo Xu, Zhen Liu, Xiaoli Ma

**Affiliations:** ^1^College of Life Science, Shandong Normal UniversityJinan, China; ^2^Research Center for Clinical and Translational Medicine, 302 Hospital of PLABeijing, China; ^3^Central Laboratory, Jinan Central Hospital Affiliated to Shandong UniversityJinan, China

**Keywords:** astrocytes, neurons, hypothalamus, gastric mucosal damage, stress

## Abstract

Restraint water-immersion stress (RWIS), a compound stress model, includes both psychological and physical stimulation. Studies have shown that neurons in the hypothalamus are involved in RWIS, but the role of astrocytes and the interactions between astrocytes and neurons in RWIS are not clear. Here, we tested our hypothesis that hypothalamus astrocytes are involved in RWIS and interact with neurons to regulate gastric mucosal damage induced by RWIS. The expression of Glial fibrillary acidic protein (GFAP) and c-Fos in the paraventricular nucleus (PVN) and supraoptic nucleus (SON) significantly increased following the RWIS. GFAP and c-Fos expression are similar in the temporal pattern, peaked at 1 h after the RWIS, then reduced gradually, and reached a maximal level again at 5 h which show “double-peak” characteristics. Intracerebroventricular administration of astroglial toxin L-a-aminoadipate (L-AA) and c-Fos antisense oligodeoxy nucleotides (ASO) both decreased RWIS-induced gastric mucosal damage. Results of immunohistochemistry assay revealed that both L-AA and ASO decreased the activation of astrocytes and neurons in the hypothalamus by RWIS. These results showed that hypothalamus neuron-astrocyte “network” involved in gastric mucosal damage induced by RWIS. This study may offer theoretical basis for some novel therapeutic strategies for RWIS-induced gastric ulcers.

## Introduction

Restraint water-immersion stress (RWIS) in rats can imitate clinical gastric lesions caused by wounds, surgery or sepsis. Therefore, RWIS is often used to study the etiopathogenesis of stress-induced gastric mucosal damage (Ai and Zhang, 1990; Ephgrave et al., 1997). Gastric function is primarily controlled by the parasympathetic nervous system through the dorsal motor nucleus of the vagus nerve (DMV; Andrews and Sanger, 2002; Hayakawa et al., 2003). The paraventricular nucleus (PVN) and supraoptic nucleus (SON) are the most prominent nuclei in the anterior hypothalamus and may be stimulus-dependent (Palkovits, 2000; Briski and Gillen, 2001). Functional and anatomical connections among PVN neurons, the lower brainstem and spinal cord have been established. Parvocellular oxytocin (OT) cells in the PVN project mainly to the nucleus of solitary tract (NTS), the DMV and the intermediolateral cell column of the spinal cord (Kannan and Yamashita, 1985; Luiten et al., 1985). These neurons are believed to play a major role in gastric reflexes.

Astrocytes are the most numerous neuroglial cells in the central nervous system (CNS). In the last few decades, evidence from a series of studies has demonstrated that astrocytes and neurons share transporters and receptors, and astrocytes influence synaptic signaling as well as synchronizing neuronal activity and calcium dynamics by releasing gliotransmitters (Lavialle et al., 2011). Most of the research on the neurobiology of stress has been exclusively focused on neurons (Hayakawa et al., 2003; Furlong et al., 2014). However, recent findings suggested that astrocytes can directly sense and eventually change their morphology or functionality in response to the stress (Young et al., 2008; Kirby et al., 2013). Our study has reported that astrocytes in the brainstem are involved in RWIS-induced gastric mucosal damage (Sun et al., 2016). Due to the close relationship between the hypothalamus and brainstem in structure and function, it is possible that hypothalamus astrocytes participate in RWIS or interact with neurons to regulate gastric mucosal damage induced by the RWIS model.

c-Fos has been used as a functional anatomical mapping tool to identify cells and serves as a marker of neuronal activity (Furlong et al., 2014). Astrocytes have the potential to play important roles in various physiological or noxious stimuli with increased Glial fibrillary acidic protein (GFAP) expression. Therefore, GFAP is commonly used as an astrocytic activation marker (Takahashi et al., 2006; Sechi et al., 2010). L-a-aminoadipate (L-AA) is used to deactivate astrocytes based on the fact that disruption of the astrocytic network in the amygdala is confined to astrocytes (Khurgel et al., 1996; Wang et al., 2009). A c-Fos antisense oligodeoxy nucleotides (ASO) was observed when a c-Fos protein product was knocked down (Lee et al., 2004). Therefore, L-AA and ASO were employed in this study to shut down activated astrocytes and hypothalamus neurons, respectively.

In this study, we examined the time course of astrocyte and neuron activation in the PVN and SON along with RWIS-induced gastric mucosal damage by using GFAP and c-Fos expression, respectively. In addition, the potential “network” between astrocytes and neurons was investigated with intracerebroventricular injection of L-AA or c-Fos ASO.

## Materials and Methods

### Preparation of Animals

Male Wistar rats weighing 220–250 g were purchased from the Experimental Animal Center of Shandong University. Before the stress procedure, the rats were housed two per cage under a normal 12:12 h light/dark cycle (light on at 6:00 and off at 18:00) at 22 ± 2°C ambient temperature with food and water available *ad libitum*.

RWIS was carried out after the rats were starved for 24 h with free access to water. All experiments were carried out in accordance with the guidelines of the International Association for the Study of Pain (Zimmermann, 1986) and approved by the Experimental Animal Ethics Committee of Shandong Normal University (Jinan, China).

### Stress Protocols

The animals were randomly divided into six groups according to the duration of RWIS: 0, 0.5, 1, 2, 3, and 5 h (*n* = 6 per group). The rats were lightly anesthetized by ether inhalation and the limbs of each rat were bound gently and securely on a wooden plate using medical adhesive tape. After the rats regained consciousness, they were vertically immersed in water maintained at 21°C to the level of the xiphoid for 0.5, 1, 2, 3, and 5 h. Rats in the control group were neither bound to the wooden board nor immersed in water. The experiment was performed between 8:00 and 13:00 to decrease the effect of diurnal variations on GFAP and c-Fos expression.

### Administration of Drugs

The phosphorothioate-modified c-Fos ASO and the L-AA were obtained from Sangon Biotechnology Co. (Shanghai, China) and Sigma (St. Louis, MO, USA), respectively. The rats were randomly assigned to three groups: (1) ASO group with c-Fos ASO (50 μg dissolved in 10 μl saline,) injected into the lateral ventricle (*n* = 6); (2) L-AA group with an intracerebroventricular injection of L-AA at a dose of 100 nmol in 10 μl saline; and (3) Saline group with an intracerebroventricular administration of normal saline (NS, 10 μl, *n* = 6). Stereotaxic coordinates were: 0.8 mm posterior, 1.8 mm right lateral to the Bregma, and 3.8 mm ventral from the Bregma, according to the Paxinos and Watson’s brain atlas. For each injection, the solution was slowly injected within 5 min. The needle was kept in place for 5 min to lower pressure inside the tube. The rats received the injection and then were stressed with RWIS for 1 h. Because expression of GFAP and c-Fos peaked at 1 h of RWIS, the 1 h RWIS time point was used for the drug experiments.

### Tissue Preparation

At the end of the experiments, the rats were sacrificed with an overdose of pentobarbital sodium and perfused through the left cardiac ventricle with approximately 200 ml 0.01 mol/L phosphate buffered saline (PBS, pH 7.4) followed by approximately 300 ml 4% paraformaldehyde in phosphate buffer (0.1 M, pH 7.4). The brain was removed after the end of perfusion and post-fixed at 4°C for 4 h. Next, the brains were cryoprotected by infiltration with 20% sucrose in 0.1 mol/L phosphate buffer at 4°C for 48 h.

### Immunohistochemistry

Frozen coronal sections were cut 30 μm thick and collected serially in two dishes. Each dish contained a complete set of serial sections that were processed for immunohistochemistry. The sections were blocked with 2% goat serum in 0.01 MPBS containing 0.3% Triton X-100 for 1 h at room temperature (RT). The GAFP-immunoreactive (GAFP-IR) and c-Fos-immunoreactive (c-Fos-IR) sections were incubated overnight at 4°C with mouse anti-GFAP (1:500; Chemicon, Temecula, CA, USA) and rabbit anti-Fos (1:1000; Santa Cruz Biotechnology, Santa Cruz, CA, USA), respectively. After washing the samples three times with PBS for 10 min, the sections were incubated for 1 h in the secondary antibodies at RT. The specificities of the staining were tested on the sections in the second dish by omitting the primary antibodies. No immunoreactive outcomes were found on the sections.

### Evaluation of Gastric Mucosal Damage

The rats were sacrificed after the stress protocols were completed. Their stomachs were removed and incised along the greater curvature of stomach, then rinsed with normal saline. Gastric mucosal lesions were identified with a magnifying lens. The erosion index (EI) was measured according to the methods of Guth (1992). Scores were made according to the length of the lesion: the length ≤1 mm as 1 score, 1 mm < the length ≤2 mm as 2 score, and the others were deduced in turn. The score was multiplied by 2 when the width of the lesion was larger than 1 mm. The cumulative scores of all lesions in a rat served as the EI of the rat.

### Evaluation of Immunostaining

Brain sections were observed under identical conditions with a BX51 Olympus microscope (Olympus Corporation, Japan). The nomenclature and nuclear boundaries defined in the rat brain stereotaxic atlas of Paxinos and Watson (Picciotto and Kenny, 2013) were used. For quantitative assessment, Image-Pro Plus 6.0 (Media Cybernetics Inc, Rockville, MD, USA) was used to quantify GFAP-IR astrocytes and the c-Fos expression in PVN and SON. The pictures taken of GFAP-IR astrocytes were magnified 200 times, and pictures taken of the c-Fos-IR neurons were magnified 100 times. GFAP-positive astrocytes and c-Fos positive neurons were counted in the hypothalamus (PVN and SON) during RWIS. The number of immunoreactive astrocytes and neurons were counted from three inconsecutive sections per animal, and the average values of the immunoreactive astrocytes and neurons in 0.01 mm^2^ were reported as the measure of immunoreactivity.

### Statistical Analysis

All of the values were reported as the mean ± SEM. The statistical procedures were performed with the SPSS13.0 software (SPSS, Chicago, IL, USA). Comparisons between two groups were conducted by using *T*-tests or a repeated measures one-way analysis of variance (ANOVA) with a Dunnett’s post-test. *P* < 0.05 was considered to be statistically significant.

## Results

### RWIS Induced Gastric Mucosal Damage

**Figure 1:**
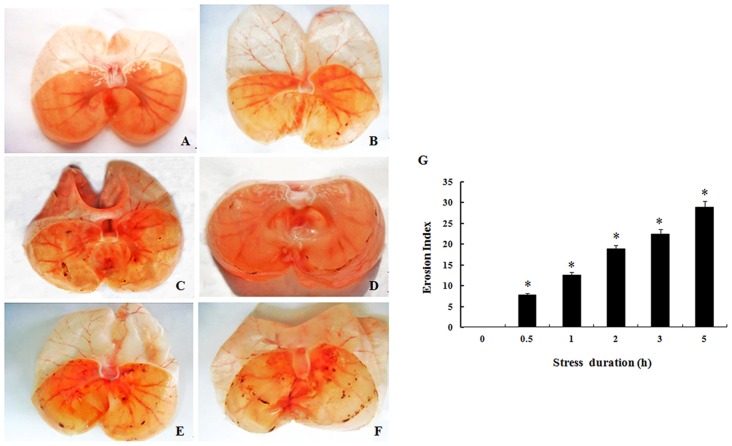
**Gastric mucosal damage induced by Restraint water-immersion stress (RWIS). (A–F)** Gastric mucosal damage at 0 **(A)**, 0.5 **(B)**, 1 **(C)**, 2 **(D)**, 3 **(E)** and 5 h **(F)** of RWIS. **(G)** Erosion index (EI) was increased time-dependently during RWIS. The data are presented as the mean ± SEM (*n* = 6). * vs. control group.

Macroscopic analysis showed that there was no gastric mucosal damage in the control group (0 h). Compared to the sham group, scattered spots or lineal hemorrhage and lesions were observed in the mucosa in the 0.5, 1, 2, 3, and 5 h groups under continuous stress, which suggests there is a significant effect of RWIS time on the erosion indices (Figures 1A–G).

### GFAP Expression in the Hypothalamus of the RWIS Rats

**Figure 2:**
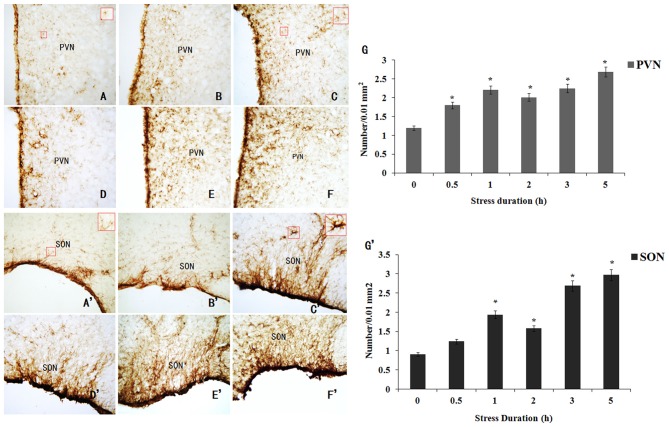
**Immunohistochemical staining of Glial fibrillary acidic protein (GFAP)-immunoreactive (IR) astrocytes in the paraventricular nucleus (PVN) and supraoptic nucleus (SON) induced by RWIS. (A–F)** GFAP-IR astrocytes in the PVN induced by RWIS at 0 **(A)**, 0.5 **(B)**, 1 **(C)**, 2 **(D)**, 3 **(E)** and 5 h **(F)** of RWIS (200×). In panel **(A,C)**, GFAP-IR astrocytes in a rectangle were magnified to a higher magnification (400×). **(G)** The number of GFAP-IR astrocytes in the PVN was quantified. **(A’–F’)** GFAP-IR astrocytes in the SON induced by RWIS at 0 **(A)**, 0.5 **(B)**, 1 **(C)**, 2 **(D)**, 3 **(E)** and 5 h **(F)** of RWIS (200×). In panel **(A’,C’)**, GFAP-IR astrocytes in a rectangle were magnified to a higher magnification (400×). **(G’)** The number of GFAP-IR astrocytes in the SON was quantified. *P < 0.05 compared to control group.

During RWIS treatment (0.5, 1, 2, 3, and 5 h), the number of GFAP positive astrocytes in the PVN and SON increased. Compared to the 0 h group, the GFAP-IR astrocytes in the RWIS groups significantly increased in the PVN and SON (Figures 2A–F,A’–F’). A time-course study showed the GFAP expression in the PVN significantly increased at 0.5 h (Figure 2G). Whereas, the GFAP-positive cell number in SON began to increase, but not significantly, 0.5 h after the stress. The GFAP expression in the PVN and SON demonstrated a double-peak in which expression peaked at 1 h after administration of the RWIS then reduced at 2 h and reached a maximal level again at 4–5 h (Figure 2G’).

### Hypothalamic c-Fos Expression in the RWIS Rats

**Figure 3:**
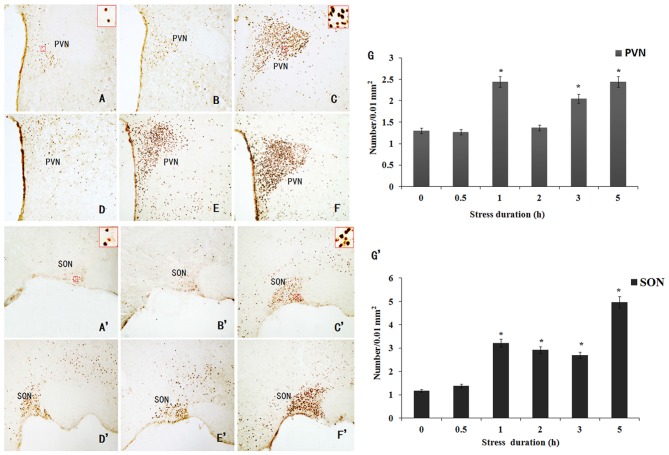
**Immunohistochemical staining of Fos-IR neurons in the PVN and SON induced by RWIS. (A–F)** Fos-IR neurons in the PVN induced by RWIS at 0 **(A)**, 0.5 **(B)**, 1 **(C)**, 2 **(D)**, 3 **(E)** and 5 h **(F)** of RWIS (100×). In panel **(A,C)**, Fos-IR neurons in a rectangle were magnified to a higher magnification (400×). **(G)** The number of Fos-IR neurons in the PVN was quantified. **(A’–F’)** Fos-IR neurons in the SON induced by RWIS at 0 **(A)**, 0.5 **(B)**, 1 **(C)**, 2 **(D)**, 3 **(E)** and 5 h **(F)** of RWIS (100×). In panel **(A’,C’)**, Fos-IR neurons in a rectangle were magnified to a higher magnification (400×). **(G’)** The number of Fos-IR neurons in the SON was quantified. *P < 0.05 compared to control group.

Neuronal activation was evaluated on the basis of c-Fos-immunoreactivity, which appeared as a brown deposit in the cell nucleus (Figure 3). Compared to the control, the c-Fos-IR neurons in the RWIS groups were significantly increased in the PVN and SON. The temporal pattern of c-Fos expression in the PVN was similar to that of the SON. The c-Fos expression in the PVN and SON peaked at 1 h after the RWIS then reduced gradually at 2–3 h but remained higher compared to the 0 h group (*P* < 0.05) and reached a maximal level again at 5 h (Figures 3A–G,A’–G’). C-Fos expression in the PVN and SON also presented with “double-peak” characteristics.

### c-Fos ASO or L-AA Treatment Lessened RWIS-Induced Gastric Damage and GFAP Expression in the Hypothalamus

**Figure 4:**
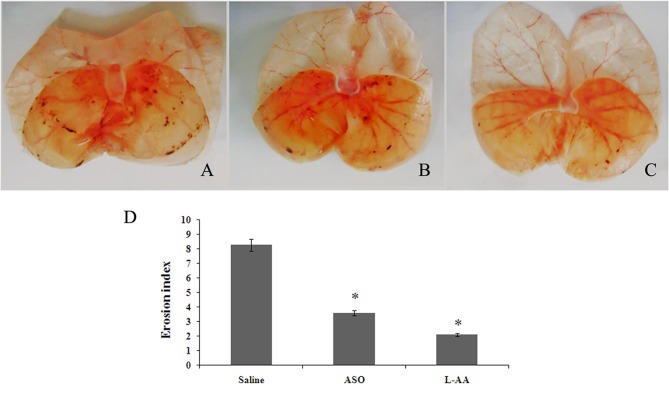
**Effects of L-a-aminoadipate (L-AA) or antisense oligodeoxy nucleotides (ASO) on RWIS-induced gastric mucosal damage. (A)** RWIS rats treated with saline showed gastric mucosal damage. **(B)** Administration of c-Fos ASO reversed the gastric mucosal damage. **(C)** Administration of L-AA lessened the gastric mucosal damage. **(D)** EI is presented as the mean ± SEM (*n* = 6). *P < 0.05 compared to the Saline group.

**Figure 5:**
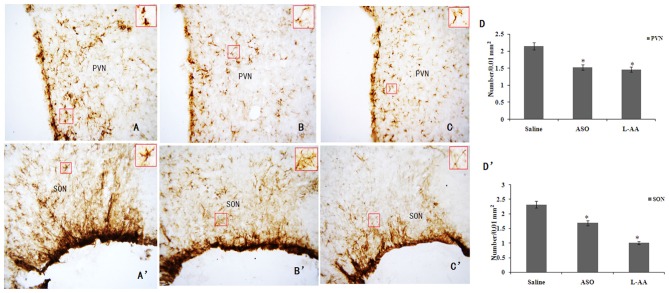
**Effects of ASO or L-AA on RWIS-induced hypothalamic GFAP expression. (A–C)** GFAP expression in the PVN in saline, ASO and L-AA groups (200×). **(B,A’–C’)** GFAP expression in the SON in saline, ASO and L-AA groups (200×). **(D,D’)** The number of GFAP-IR neurons in the PVN and SON was quantified. *P < 0.05 compared to control group.

RWIS rats treated with saline demonstrated significant gastric mucosal damage (Figure 4A). C-Fos ASO or L-AA treatment significantly reduced gastric mucosal damage (Figures 4B,C). Therefore, an intracerebroventricular injection of c-Fos ASO or L-AA in RWIS rats significantly depressed the extent of gastric mucosal damage (Figure 4D). This change was accompanied by an attenuating effect on the RWIS-enhanced hypothalamus (PVN and SON) GFAP expression (Figure 5). L-AA treatment had an inhibitory effect on the enhanced GFAP compared to the saline control in the hypothalamus (PVN and SON; Figures 5C,C’). Interestingly, C-Fos ASO treatment also suppressed RWIS-induced GAFP upregulation in the hypothalamus (PVN and SON; Figures 5B,B’).

### c-Fos ASO or L-AA Treatment in the RWIS Rats Reduced Hypothalamic c-Fos Expression

**Figure 6:**
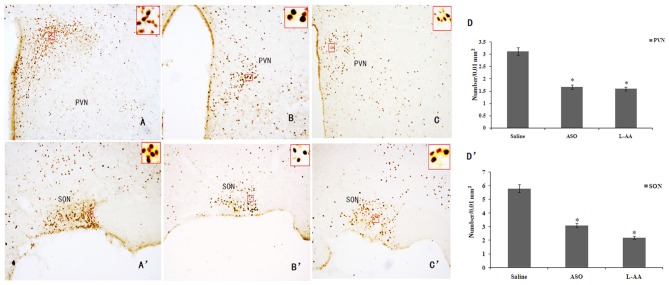
**Effects of ASO or L-AA on RWIS-induced hypothalamic c-Fos expression. (A–C)** c-Fos expression in the PVN in saline, ASO and L-AA groups (100×). **(A’–C’)** c-Fos expression in the SON in saline, ASO and L-AA groups (100×). **(D,D’)** The number of Fos-IR neurons in the PVN and SON was quantified. *P < 0.05 compared to control group.

In addition to the effect of c-Fos ASO or L-AA treatment on hypothalamic GFAP expression in the RWIS rats, c-Fos ASO or L-AA treatment also significantly inhibited RWIS-induced hypothalamic c-Fos protein expression compared to the saline group (Figure 6). The c-Fos level in the ASO group was inhibited compared to c-Fos expression in the saline group (Figures 6B,B’). Meanwhile, L-AA treatment also suppressed c-Fos expression in the PVN and SON (Figures 6C,C’). An intracerebroventricular injection of c-Fos ASO or L-AA had a protective effect on RWIS-induced gastric mucosal damage.

## Discussion

The study of RWIS-induced gastric mucosal damage may be recognized at psychological, physiological, organic, cellular and molecular levels (Ueyama et al., 1998; Guo et al., 2012). However, the mechanism underlying this damage is far from clear. In this study, we assessed hypothalamic astrocytes involved in RWIS-induced gastric mucosal damage in rats and examined interactions with hypothalamic neurons during this process. In this study, the main findings were that astrocytes and neurons in the hypothalamus (PVN and SON) are involved in the effect of stress on gastrointestinal function, inhibiting either neuronal or astrocytic activity in the hypothalamus prevented RWIS-induced gastric mucosal damage, and a neuron-astrocyte interaction in the hypothalamus occurs under RWIS conditions.

### RWIS Enhanced Neuronal and Astrocytic Activations in the Hypothalamus

The PVN and SON in the hypothalamus are two of the most important nuclei and are fundamental in homeostatic regulation. Prior studies suggest phenotypic changes occur in the hypothalamus (PVN and/or SON) in response to acute and chronic stress, including elevations in vasopressin (VP) and OT and/or corticotrophin releasing factor (CRF) gene expression (Aguilera et al., 2008; Blume et al., 2008). Alterations of gene expression in the PVN and SON have important functional roles in the regulation of the hypothalamo-neurohypophysial system (HNS) and the hypothalamic–pituitary–adrenal (HPA) axis during stress. Acute and chronic stress may lead to changes in the VP and OT signature and be connected to the amount of neuroendocrinological disturbances (Al-Barazanji et al., 2001). These changes may lead to the dysregulation of the autonomic nervous system under many conditions. The large magnocellular neurons in the PVN and SON produce mainly VP and OT as neurotransmitters, which have connections to gastric functions (Flanagan et al., 1992; Lu et al., 2011).

In the present study, immunohistochemical assays of c-Fos expression suggested there was higher c-Fos immunoreactivity in the PVN and SON in the stressed rats compared to the control group. The non-stressed basal conditions do not significantly increase c-Fos expression and show no c-Fos positive cells or scattered immunoreactive cells. The result is consistent with a previous study that indicated that restraint stress caused c-Fos expression in the hypothalamus (Amemiya et al., 2005; Pace et al., 2009). After RWIS, neurons were activated in PVN and SON and indicated that PVN and SON might be involved in gastric dysfunction induced by RWIS, such as gastric hypermotility, and contribute to stress-induced gastric mucosa damage.

Meanwhile, we found that the time course of c-Fos expression induced by RWIS was different from expression in other observations. Acute challenge leads to c-Fos expression within a few minutes and peaks between 30 and 60 min. The maximal amount of c-Fos protein occurs between 1 h and 3 h then gradually decreases from the cell nucleus by 4–6 h after stimuli (Sawchenko et al., 1996; Ziółkowska et al., 2015). These results were consistent with our previous observations that the peak of stress-induced c-Fos expression in the brainstem occurred at 1 h after the RWIS and decreased following stress at 2–5 h (Sun et al., 2016). Yet our present results show that the c-Fos expression in the PVN and SON peaked at 1 h after the RWIS then reduced gradually at 2–3 h before reaching a maximal level again at 5 h, which suggests a biphasic pattern of immunoreactivity. This indicted that the c-Fos expression spatial patterns during RWIS may be different between the brainstem and the hypothalamus.

It has been demonstrated that the early wave of c-Fos expression is part of a rapid pulse of increased gene transcription involving a broad functional repertoire of molecules, including transcription factors, growth factors and signal transduction molecules (Lanahan and Worley, 1998) acting as a response to exogenous stimulation that are involved in the growth and differentiation of neurons and the modulation of repair after injury. The second peak of c-Fos expression induced by RWIS is unexpected. There are studies demonstrating that a delayed expression of c-Fos occurred after a single challenge (Bing et al., 1997), that a peak of c-Fos expression appeared in 4–7 days in the brain and was connected with excitotoxic cell death (Smeyne et al., 1993) and that the biphasic induction of c-Fos expression was caused by diffuse brain injury (Zheng et al., 2014).

Much of the previous research implies that transient c-Fos expression is induced by acute challenges and is not occurring in response to chronic stress. Later studies showed that c-Fos positive neurons were persistently detected in the SON and PVN of rats after drinking a hypertonic NaCl solution instead of water (Miyata et al., 1994), which suggests that chronic stress led to sustained c-Fos expression. It is possible that persistent c-Fos expression after chronic stress incorporates neural components as well as large and gradual changes in the response of those components to stimuli during development and/or neuronal plasticity (Miyata et al., 1994). In agreement with these prior results, ongoing plasticity through late expression of “plasticity genes”, such as c-Fos, can increase the capacity to process and store information (Fusi and Abbott, 2007). Therefore, in this study, the second wave of c-Fos expression may increase the transcription of other genes such as AVP or the OT gene and be associated with an event such as RWIS. Numerous pioneering studies showed the SON and PVN involved in neurosecretory and autonomic control had a highly intact neuro-glial microenvironment in which a single astrocyte can encompass thousands of synapses and allow for direct interaction with neurons. Therefore, astrocytes act as an important partner with neurons by regulating neuron-neuron interactions as well as neuronal synaptic transmissions and plasticity. However, the neuro-glial microenvironment undergoes an impressive structural remodeling in response to a variety of stimuli (Oliet et al., 2008; Tasker et al., 2012). There is substantial evidence that chronic and acute stress can induce changes in the morphology and function of astrocytes, which may impair brain function and ultimately form part of the pathophysiology of stress-related disorders (Feng et al., 2010). Therefore, it is important to think about the role that astrocyte-induced plasticity may play in the behavioral sequelae of stress. Some studies of chronic stress models showed that antidepressant drugs such as fluoxetine and clomipramine prevent stress-induced astrocyte changes and might normalize stress-induced behavioral changes (Czéh et al., 2006; Shen et al., 2012). However, the astrocytes within the PVN and SON implicated in RWIS were not investigated prior to the current study.

In recent years, it has been demonstrated that GFAP is expressed mainly in astrocytes and is a valuable marker for these cells because it is absent in neurons (Verwer et al., 2007). Upregulation of GFAP expression in response to stimulation can be studied with an immunocytochemical method (Armstrong et al., 2005). In this study, we observed RWIS-induced GFAP expression in some astrocytes in the PVN and SON. For the different durations of RWIS (0.5, 1, 2, 3, 5 h), robust GFAP expression in the PVN and SON showed a double-peak characteristic in which expression peaked at 1 h after RWIS then reduced and reached a maximal level again at 4–5 h, These results suggest that a hypothalamic neuron-astrocyte network exists and plays a role in RWIS-induced gastric mucosal damage.

### Interactions of Neurons and Astrocytes Contribute to RWIS-Induced Gastric Mucosal Damage

The number and/or function of astrocytes would affect the integrity, activity and behavioral output of a neuron-astrocyte network (Seifert et al., 2006; Halassa et al., 2007). However, astrocytes cannot directly generate action potentials alone. Our hypothesis is that the activated astrocytes and neurons interact to contribute to the gastric mucosal damage after RWIS.

To test the hypothesis, it was necessary to downregulate the activated neurons or astrocytes to show how they contributed to the gastric mucosal damage. In this study, an intracerebroventricular injection of c-Fos ASO or L-AA, respectively, was used to inhibit activated neurons or astrocytes in the PVN and SON induced by the RWIS. Decreased expression of c-Fos or GFAP was accompanied by an early stage RWIS-induced gastric mucosal damage. Notably, we observed that c-Fos ASO suppressed astrocytic activation after RWIS. It is also interesting to note that the astroglial toxin L-AA not only inhibited the GFAP expression but also blocked the c-Fos expression, which suggests that RWIS activates astroglial cells and releases unknown factors, which may modulate the activity of neurons in RWIS. Our results showed RWIS-induced c-Fos expression, which indicates a heightened activity of neurons within the PVN and SON. Moreover, activated neurons release a variety of neurotransmitters that activate astrocytes. Activated astrocytes also produce gliotransmitters (i.e., ATP) that have an important role in information processing and influence neuronal activity in the SON/PVN, which contributes to RWIS-induced gastric mucosal damage.

In summary, we provide evidence that hypothalamic astrocytes were activated and interacted with neuronal activation in response to RWIS-induced damage. Breaking this “neuron-astrocyte” interaction may help to develop novel gastric ulcer therapeutic strategies.

## Author Contributions

HS, RL and XM: conception and design of the experiments. HS, RL, SX, ZL and XM: collection, analysis and interpretation of data. HS, RL and XM: drafting the article or revising it critically for important intellectual content. All persons designated as authors qualify for authorship, and all the authors approved the final version of the manuscript for publication.

## Funding

This research was supported by grants from the National Natural Science Foundation of China (No. 31672286), the Natural Science Foundation of Shandong Province, China (ZR2013CM010 and ZR2012HM061).

## Conflict of Interest Statement

The authors declare that the research was conducted in the absence of any commercial or financial relationships that could be construed as a potential conflict of interest.
